# How Parental Predictors Jointly Affect the Risk of Offspring Congenital Heart Disease: A Nationwide Multicenter Study Based on the China Birth Cohort

**DOI:** 10.3389/fcvm.2022.860600

**Published:** 2022-06-03

**Authors:** Man Zhang, Yongqing Sun, Xiaoting Zhao, Ruixia Liu, Bo-Yi Yang, Gongbo Chen, Wangjian Zhang, Guang-Hui Dong, Chenghong Yin, Wentao Yue

**Affiliations:** ^1^Central Laboratory, Beijing Obstetrics and Gynecology Hospital, Capital Medical University, Beijing, China; ^2^Beijing Maternal and Child Health Care Hospital, Beijing, China; ^3^Prenatal Diagnosis Center, Beijing Obstetrics and Gynecology Hospital, Beijing Maternal and Child Health Care Hospital, Capital Medical University, Beijing, China; ^4^Guangzhou Key Laboratory of Environmental Pollution and Health Risk Assessment, Guangdong Provincial Engineering Technology Research Center of Environmental and Health Risk Assessment, Department of Preventive Medicine, School of Public Health, Sun Yat-sen University, Guangzhou, China; ^5^Department of Medical Statistics, School of Public Health, Sun Yat-sen University, Guangzhou, China

**Keywords:** congenital heart disease, risk factors, web-based nomogram, prediction, China birth cohort

## Abstract

**Objective:**

Congenital heart disease (CHD) is complex in its etiology. Its genetic causes have been investigated, whereas the non-genetic factor related studies are still limited. We aimed to identify dominant parental predictors and develop a predictive model and nomogram for the risk of offspring CHD.

**Methods:**

This was a retrospective study from November 2017 to December 2021 covering 44,578 participants, of which those from 4 hospitals in eastern China were assigned to the development cohort and those from 5 hospitals in central and western China were used as the external validation cohort. Univariable and multivariable analyses were used to select the dominant predictors of CHD among demographic characteristics, lifestyle behaviors, environmental pollution, maternal disease history, and the current pregnancy information. Multivariable logistic regression analysis was used to construct the model and nomogram using the selected predictors. The predictive model and the nomogram were both validated internally and externally. A web-based nomogram was developed to predict patient-specific probability for CHD.

**Results:**

Dominant risk factors for offspring CHD included increased maternal age [odds ratio (OR): 1.14, 95% CI: 1.10–1.19], increased paternal age (1.05, 95% CI: 1.02–1.09), maternal secondhand smoke exposure (2.89, 95% CI: 2.22–3.76), paternal drinking (1.41, 95% CI: 1.08–1.84), maternal pre-pregnancy diabetes (3.39, 95% CI: 1.95–5.87), maternal fever (3.35, 95% CI: 2.49–4.50), assisted reproductive technology (2.89, 95% CI: 2.13–3.94), and environmental pollution (1.61, 95% CI: 1.18–2.20). A higher household annual income (100,000–400,000 CNY: 0.47, 95% CI: 0.34–0.63; > 400,000 CNY: 0.23, 95% CI: 0.15–0.36), higher maternal education level (13–16 years: 0.68, 95% CI: 0.50–0.93; ≥ 17 years: 0.87, 95% CI: 0.55–1.37), maternal folic acid (0.21, 95% CI: 0.16–0.27), and multivitamin supplementation (0.33, 95% CI: 0.26–0.42) were protective factors. The nomogram showed good discrimination in both internal [area under the receiver-operating-characteristic curve (AUC): 0.843] and external validations (development cohort AUC: 0.849, external validation cohort AUC: 0.837). The calibration curves showed good agreement between the nomogram-predicted probability and actual presence of CHD.

**Conclusion:**

We revealed dominant parental predictors and presented a web-based nomogram for the risk of offspring CHD, which could be utilized as an effective tool for quantifying the individual risk of CHD and promptly identifying high-risk population.

## Introduction

Congenital heart disease (CHD) is the most common structural birth defect with an incidence rate of 6–12 per 1,000 newborns (1–4), and its estimated prevalence is about 19–20 per 1,000 newborns when the bicuspid aortic valve is included (5, 6). In China, a national study reported an overall CHD prevalence of 9 per 1,000 newborns (7), and it remains the leading non-infectious cause of death in infancy and childhood despite tremendous progress in surgical therapies (8). CHD imposes a substantial burden on survivors, families, and societies. It is of critical importance to identify dominant risk factors for CHD, and to predict the individual risk of CHD, in order to provide more exhaustive examinations and preventive interventions to pregnant women with higher-risk fetuses for CHD.

Congenital heart disease is complex in its etiology, although much evidence revealed its genetic causes (9, 10), and genetic defects were not detected in many CHD patients. Therefore, non-genetic factors should still be well considered. In recent years, substantial epidemiological evidence has demonstrated that non-genetic factors such as social determinants of health and adverse healthy lifestyle also contribute to abnormal cardiovascular development in fetuses, leading to cardiovascular malformation. The dominant factors of CHD could be divided into six main categories, namely (1) sociodemographic factors, including parental age (11, 12), household annual income and education level (13); (2) adverse lifestyle (14); (3) maternal diseases, including pre-pregnancy diabetes (15) and fever (16); (4) environmental factors such as secondhand smoke exposure (17) and environmental pollution (18); (5) supplements such as folic acid and multivitamin (19); and (6) mode of conception (20). Previous research mainly focused on the association between limited predictors and the risk of CHD, and some conclusions involving maternal fever (16, 21), maternal folic acid, and multivitamin supplementation (19, 22, 23) have been inconsistent. Moreover, the assessment in most of the prior studies was based on data from a single or limited number of medical institutions and was not internally or externally validated, which may have influenced the generalizability and robustness of the results. To the best of our knowledge, no study has been reported on the combined effect of the above factors on the risk of offspring CHD till present. Multicenter collaboration, rigorous study design, and adequate validation are important elements in the effort to develop a reliable prediction tool for offspring CHD based on multiple factors.

Therefore, the aim of this study was to (a) identify and evaluate the dominant predictors of offspring CHD and (b) develop a predictive model and web-based nomogram to directly indicate the probability of offspring CHD occurrence of each pregnant woman.

## Materials and Methods

### Study Design and Participants

**FIGURE 1:**
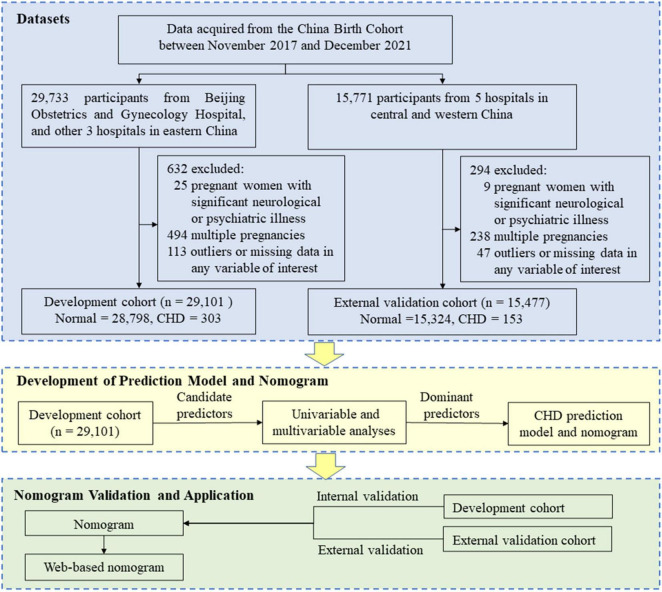
Flowchart of the study. CHD, congenital heart disease.

This was a retrospective study and we used data from the China Birth Cohort Study (CBCS), which is a population-based birth defect surveillance system in China from November 2017 to December 2021 (24). The CBCS was conducted in 38 research sites in 17 provinces, cities, autonomous regions, and municipalities covering most areas of China. We obtained data access permissions for 9 hospitals among them. Data of each participant including birth outcome, demographic characteristics, lifestyle behaviors, environmental pollution, maternal disease history, and the current pregnancy information were required to be reported by trained obstetricians, sonographers, and pediatricians for the same CBCS system. To build a robust nomogram, participants from four hospitals in eastern China, including Beijing Obstetrics and Gynecology Hospital, were used as a development cohort. Participants from five hospitals in central and western China were used as an external validation cohort. All participants’ data of the above nine hospitals were collected. For the development cohort, 29,733 participants were initially recruited. Of these, 25 participants were excluded because of significant neurological or psychiatric illness of pregnant women (the accuracy of questionnaires could not be guaranteed), 494 multiple pregnancies were excluded since etiology of CHD may be different between singleton and multiple pregnancies (18), and a further 113 participants were excluded due to outliers or missing data in any variable of interest. Thus, 29,101 participants (28,798 normal + 303 with CHD) were retrieved for inclusion in the development cohort. At the same time, 15,477 participants (15,324 normal + 153 with CHD) were used as the external validation cohort using the same criteria as for the development cohort (Figure 1 and Supplementary Table 1). The sensitivity analysis that excluded participants with missing data yielded results similar to those presented (data not shown). This study was guided by the Transparent Reporting of a Multivariable Prediction Model for Individual Prognosis or Diagnosis (TRIPOD) criteria (Supplementary Table 2) (25).

### Primary Outcome and Predictors

Our primary end point was the presence or absence of offspring CHD. CHD cases included livebirths, stillbirths, and late abortions who were diagnosed as CHD for more than 17 weeks of gestation. All cases were evaluated by a trained obstetrician, pediatrician, or cardiologist before hospital discharge or within 3 days after birth. Two echocardiography experts independently examined the echocardiographic images of each CHD case, and they were blinded to the outcome of each other. If there was still a dispute, computed tomography, cardiac catheterization, or autopsy were used to further determine a CHD case. All CHD cases were classified and recorded based on the International Classification of Diseases, Tenth Revision Modification (ICD-10-CM).

We identified potential predictors using a literature search. The following candidate predictors were selected from our standard and structured questionnaires: parental age, parental ethnicity (Han or Minority), parental first-trimester body mass index (BMI), household annual income (< 100,000 CNY or 100,000–400,000 CNY or > 400,000 CNY), parental education level (≤ 12 years or 13–16 years or ≥ 17 years), parental smoking (yes or no), maternal secondhand smoke exposure (yes or no), parental drinking (yes or no), maternal pre-pregnancy diabetes (yes or no), maternal fever (yes or no), maternal folic acid supplementation (yes or no), maternal multivitamin supplementation (yes or no), mode of conception (natural conceived or assisted reproductive technology), and environmental pollution (yes or no).

The height and weight of the participants were obtained through an accurate measurement. The standing height was measured to the nearest 0.1 cm using a stadiometer (26). The value of weight was accurately measured using an electronic scale (BW-150; UWE, Beijing, China) with participants wearing light clothes, no shoes, and empty pockets (27). The BMI was calculated as the weight in kilograms divided by the square of the height in meters. Parental smoking or drinking was defined as mothers or fathers who smoked at least one cigarette per day or drank alcohol once a week for over 6 months (28). Maternal secondhand smoke exposure was defined as non-smokers being exposed to another person’s tobacco smoke for at least 15 min daily for more than 1 day per week (29). Pre-pregnancy diabetes of pregnant women was defined as type 1 or type 2 diabetes diagnosed before pregnancy (15). Maternal fever was defined as body temperature exceeding 38°C or self-reported fever experience in the first trimester (16). Environmental pollution of this study was defined as the presence of smelly water ditches, garbage stations, or coal-fired factories within 100 m of the residential area.

### Statistical Analyses

Data were given as a median (interquartile range) or n (%). The independent *t*-test and Mann–Whitney *U* test were used to analyze differences in continuous variables between groups, while the chi-square test and the Fisher’s exact test were used for categorical variables. The univariable and multivariable analyses were used to select the dominant predictors. The multivariable logistic regression analysis was used to construct a risk prediction model for offspring CHD, based on the selected predictors of the development cohort. The variables in the final multivariable model were selected by the bidirectional stepwise regression with the Akaike information criterion. The results were described as the odds ratio (OR) and 95% confidence interval (CI). In addition, the outliers and collinearity of multivariable regression model were assessed. A nomogram consisting of the dominant predictors was created to translate the prediction model into a visual scoring system to calculate the estimated probability of offspring CHD. Each predicator was assigned a “points” on the nomogram, based on its predictive ability for offspring CHD. High total points based on the sum of the assigned points for each predicator in the nomogram were associated with a high CHD probability. To validate the performance of the nomogram, both internal and external validations were conducted. For both of them, the discriminatory ability of the nomogram was assessed using the receiver-operating-characteristic curve (AUC); if the AUC was closer to 1, the nomogram was seen as having a good discriminatory ability (30); the calibration of the nomogram was assessed using the calibration curves. To assess possible overfitting, the Delong test was implemented to compare the AUCs. Besides, a web-based nomogram was created to directly indicate the patient-specific probability of offspring CHD occurrence. All statistical tests were performed using the R statistical software version 4.1.1^1^. The statistical significance was assumed at *P* < 0.05.

## Results

### Characteristics of Participants

The characteristics of participants in the development and external validation cohorts are presented in Table 1. In the development and external validation cohorts, the median [interquartile range (IQR)] age of maternal age was 31.0 (29.0–34.0) and 31.0 (28.0–34.0) years, respectively. The paternal age was 33.0 (30.0–36.0) and 33.0 (30.0–37.0) years, respectively. The development cohort had a similar parental first-trimester BMI, parental ethnicity, household annual income, parental smoking, maternal secondhand smoke exposure, parental drinking, maternal pre-pregnancy diabetes, mode of conception, and environmental pollution status compared with the external validation cohort. A total of 456 CHD cases were included in this study, of which the three most types were ventricular septal defect (35.75%), multiple congenital heart disease (26.10%), and Tetralogy of Fallot (13.38%) (Supplementary Table 3).

**TABLE 1:** Characteristics of study populations.

	Total (*n* = 44,578)	Development cohort (*n* = 29,101)	External validation cohort (*n* = 15,477)	*P* value
Maternal age, year	31.00 (29.00–34.00)	31.00 (29.00–34.00)	31.00 (28.00–34.00)	< 0.001
Paternal age, year	33.00 (30.00–36.00)	33.00 (30.00–36.00)	33.00 (30.00–37.00)	< 0.001
Maternal first-trimester BMI, kg/m^2^	21.22 (19.57–23.31)	21.12 (19.56–23.19)	21.26 (19.60–23.44)	< 0.001
Paternal first-trimester BMI, kg/m^2^	24.22 (22.39–26.35)	24.22 (22.30–26.27)	24.38 (22.49–26.54)	< 0.001
**Offspring congenital heart disease**				0.599
No	44,122 (98.98%)	28,798 (98.96%)	15,324 (99.01%)	
Yes	456 (1.02%)	303 (1.04%)	153 (0.99%)	
**Maternal ethnicity**				0.059
Han	41,649 (93.43%)	27,236 (93.59%)	14,413 (93.13%)	
Minority	2,929 (6.57%)	1,865 (6.41%)	1,064 (6.87%)	
**Paternal ethnicity**				0.041
Han	42,073 (94.38%)	27,513 (94.54%)	14,560 (94.08%)	
Minority	2,505 (5.62%)	1,588 (5.46%)	917 (5.92%)	
**Household annual income, CNY**				0.001
< 100,000	5,078 (11.39%)	3,431 (11.79%)	1,647 (10.64%)	
100,000–400,000	27,543 (61.79%)	17,915 (61.56%)	9,628 (62.21%)	
> 400,000	11,957 (26.82%)	7,755 (26.65%)	4,202 (27.15%)	
**Maternal education**				< 0.001
≤ 12 years	12,427 (27.88%)	9,098 (31.27%)	3,329 (21.51%)	
13–16 years	22,582 (50.66%)	14,278 (49.06%)	8,304 (53.65%)	
≥ 17 years	9,569 (21.46%)	5,725 (19.67%)	3,844 (24.84%)	
**Paternal education**				< 0.001
≤ 12 years	13,500 (30.28%)	9,313 (32.00%)	4,187 (27.05%)	
13–16 years	21,395 (48.00%)	13,841 (47.56%)	7,554 (48.81%)	
≥ 17 years	9,683 (21.72%)	5,947 (20.44%)	3,736 (24.14%)	
**Maternal smoking**				0.403
No	43,504 (97.59%)	28,387 (97.55%)	15,117 (97.67%)	
Yes	1,074 (2.41%)	714 (2.45%)	360 (2.33%)	
**Maternal secondhand smoke exposure**				0.401
No	36,208 (81.22%)	23,604 (81.11%)	12,604 (81.44%)	
Yes	8,370 (18.78%)	5,497 (18.89%)	2,873 (18.56%)	
**Paternal smoking**				0.222
No	28,587 (64.13%)	18,603 (63.93%)	9,984 (64.51%)	
Yes	15,991 (35.87%)	10,498 (36.07%)	5,493 (35.49%)	
**Maternal drinking**				0.462
No	42,782 (95.97%)	27,914 (95.92%)	14,868 (96.07%)	
Yes	1,796 (4.03%)	1,187 (4.08%)	609 (3.93%)	
**Paternal drinking**				0.998
No	32,011 (71.81%)	20,897 (71.81%)	11,114 (71.81%)	
Yes	12,567 (28.19%)	8,204 (28.19%)	4,363 (28.19%)	
**Maternal pre-pregnancy diabetes**				0.997
No	43,740 (98.12%)	28,554 (98.12%)	15,186 (98.12%)	
Yes	838 (1.88%)	547 (1.88%)	291 (1.88%)	
**Maternal fever**				< 0.001
No	39,197 (87.93%)	26,080 (89.62%)	13,117 (84.75%)	
Yes	5,381 (12.07%)	3,021 (10.38%)	2,360 (15.25%)	
**Maternal folic acid supplementation**				0.029
No	4,869 (10.92%)	3,110 (10.69%)	1,759 (11.37%)	
Yes	39,709 (89.08%)	25,991 (89.31%)	13,718 (88.63%)	
**Maternal multivitamin supplementation**				< 0.001
No	10,785 (24.19%)	7,433 (25.54%)	3,352 (21.66%)	
Yes	33,793 (75.81%)	21,668 (74.46%)	12,125 (78.34%)	
**Mode of conception**				< 0.001
Natural conceived	41,152 (92.31%)	26,990 (92.75%)	14,162 (91.50%)	
Assisted reproductive technology	3,426 (7.69%)	2,111 (7.25%)	1,315 (8.50%)	
**Environmental pollution**				0.958
No	39,099 (87.71%)	25,526 (87.72%)	13,573 (87.70%)	
Yes	5,479 (12.29%)	3,575 (12.28%)	1,904 (12.30%)	

*Results in table: Median (Q1-Q3)/N (%). CNY, China Yuan; BMI, body mass index.*

### Dominant Predictors for Offspring Congenital Heart Disease

In univariable analysis based on the data of the development cohort, the predictors associated with offspring CHD were maternal age (1.19, 95% CI: 1.15–1.22), paternal age (1.13, 95% CI: 1.11–1.15), household annual income (100,000–400,000 CNY, 0.29, 95% CI: 0.22–0.37; > 400,000 CNY, 0.16, 95% CI: 0.11–0.23), maternal education (13–16 years, 0.40, 95% CI: 0.31–0.52; ≥ 17 years, 0.38, 95% CI: 0.27–0.54), paternal education (13–16 years, 0.51, 95% CI: 0.40–0.65; ≥ 17 years, 0.38, 95% CI: 0.26–0.54), maternal secondhand smoke exposure (2.44, 95% CI: 1.93–3.09), paternal smoking (2.00, 95% CI: 1.59–2.51), maternal drinking (1.95, 95% CI: 1.27–3.00), paternal drinking (2.36, 95% CI: 1.88–2.96), maternal pre-pregnancy diabetes (3.38, 95% CI: 2.08–5.48), maternal fever (3.49, 95% CI: 2.71–4.50), maternal folic acid supplementation (0.16, 95% CI: 0.13–0.21), maternal multivitamin supplementation (0.31, 95% CI: 0.25–0.39), mode of conception (5.24, 95% CI: 4.07–6.75), and environmental pollution (2.73, 95% CI: 2.12–3.53). In contrast, parental ethnicity, parental first-trimester BMI, and maternal smoking did not show any association with offspring CHD (Table 2).

**TABLE 2:** Dominant predictors for offspring congenital heart disease.

Exposure	Univariable		Multivariable		
	OR (95% CI)	*P* value	β ^a^	OR (95% CI)	*P* value
Maternal age, year	1.19 (1.15–1.22)	<0.001	0.13	1.14 (1.10–1.19)	<0.001
Paternal age, year	1.13 (1.11–1.15)	<0.001	0.05	1.05 (1.02–1.09)	0.001
**Maternal ethnicity**					
Han	Ref				
Minority	1.03 (0.65–1.63)	0.891			
**Paternal ethnicity**					
Han	Ref				
Minority	0.53 (0.27–1.03)	0.060			
Maternal first-trimester BMI, kg/m^2^	1.01 (0.97–1.05)	0.581			
Paternal first-trimester BMI, kg/m^2^	1.02 (0.99–1.06)	0.221			
**Household annual income, CNY**					
<100,000	Ref				
100,000–400,000	0.29 (0.22–0.37)	<0.001	−0.76	0.47 (0.34–0.63)	<0.001
> 400,000	0.16 (0.11–0.23)	<0.001	−1.46	0.23 (0.15–0.36)	<0.001
**Maternal education**					
≤ 12 years	Ref				
13–16 years	0.40 (0.31–0.52)	<0.001	−0.39	0.68 (0.50–0.93)	0.015
≥ 17 years	0.38 (0.27–0.54)	<0.001	−0.14	0.87 (0.55–1.37)	0.543
**Paternal education**					
≤ 12 years	Ref				
13–16 years	0.51 (0.40–0.65)	<0.001			
≥ 17 years	0.38 (0.26–0.54)	<0.001			
**Maternal smoking**					
No	Ref				
Yes	1.51 (0.82–2.76)	0.186			
**Maternal secondhand smoke exposure**					
No	Ref				
Yes	2.44 (1.93–3.09)	<0.001	1.06	2.89 (2.22–3.76)	<0.001
**Paternal smoking**					
No	Ref				
Yes	2.00 (1.59–2.51)	<0.001			
**Maternal drinking**					
No	Ref				
Yes	1.95 (1.27–3.00)	0.002			
**Paternal drinking**					
No	Ref				
Yes	2.36 (1.88–2.96)	<0.001	0.34	1.41 (1.08–1.84)	0.013
**Maternal pre-pregnancy diabetes**					
No	Ref				
Yes	3.38 (2.08–5.48)	<0.001	1.22	3.39 (1.95–5.87)	<0.001
**Maternal fever**					
No	Ref				
Yes	3.49 (2.71–4.50)	<0.001	1.21	3.35 (2.49–4.50)	<0.001
**Maternal folic acid supplementation**					
No	Ref				
Yes	0.16 (0.13–0.21)	<0.001	−1.55	0.21 (0.16–0.27)	<0.001
**Maternal multivitamin supplementation**					
No	Ref				
Yes	0.31 (0.25–0.39)	<0.001	−1.11	0.33 (0.26–0.42)	<0.001
**Mode of conception**					
Natural conceived	Ref				
Assisted reproductive technology	5.24 (4.07–6.75)	<0.001	1.06	2.89 (2.13–3.94)	<0.001
**Environmental pollution**					
No	Ref				
Yes	2.73 (2.12–3.53)	<0.001	0.48	1.61 (1.18–2.20)	0.003

*Logit (offspring congenital heart disease) = −8.79 + 0.13 × Maternal age + 0.05 × Paternal age − 0.76 × (Household annual income = 100,000–400,000) − 1.46 × (Household annual income > 400,000) − 0.39 × (Maternal education = 13–16 years) − 0.14 × (Maternal education ≥ 17 years) + 1.06 × (Maternal secondhand smoke exposure = Yes) + 0.34 × (Paternal drinking = Yes) + 1.22 × (Maternal pre-pregnancy diabetes = Yes) + 1.21 × (Maternal fever = Yes) − 1.55 × (Maternal folic acid supplementation = Yes) − 1.11 × (Maternal multivitamin supplementation = Yes) + 1.06 × (Mode of conception = Assisted reproductive technology) + 0.48 × (Environmental pollution = Yes).*

*CNY, China Yuan; BMI, Body mass index; OR, odds ratio; CI, confidence interval; Ref, reference group.*

*^a^Unstandardized β coefficients were calculated from the multivariable logistic regression model.*

Multivariable logistic regression analysis led us to exclude paternal education, paternal smoking, and maternal drinking with *P* values higher than 0.05. Dominant risk factors for offspring CHD included increased maternal age (1.14, 95% CI: 1.10–1.19), increased paternal age (1.05, 95% CI: 1.02–1.09), maternal secondhand smoke exposure (2.89, 95% CI: 2.22–3.76), paternal drinking (1.41, 95% CI: 1.08–1.84), maternal pre-pregnancy diabetes (3.39, 95% CI: 1.95–5.87), maternal fever (3.35, 95% CI: 2.49–4.50), assisted reproductive technology (2.89, 95% CI: 2.13–3.94), and environmental pollution (1.61, 95% CI: 1.18–2.20). A higher household annual income (100,000–400,000 CNY, 0.47, 95% CI: 0.34–0.63; > 400,000 CNY, 0.23, 95% CI: 0.15–0.36), higher maternal education level (13–16 years, 0.68, 95% CI: 0.50–0.93; ≥ 17 years, 0.87, 95% CI: 0.55–1.37), maternal folic acid supplementation (0.21, 95% CI: 0.16–0.27), and maternal multivitamin supplementation (0.33, 95% CI: 0.26–0.42) were found to be protective factors for offspring CHD (Table 2).

### Development of Prediction Model and Nomogram

**FIGURE 2:**
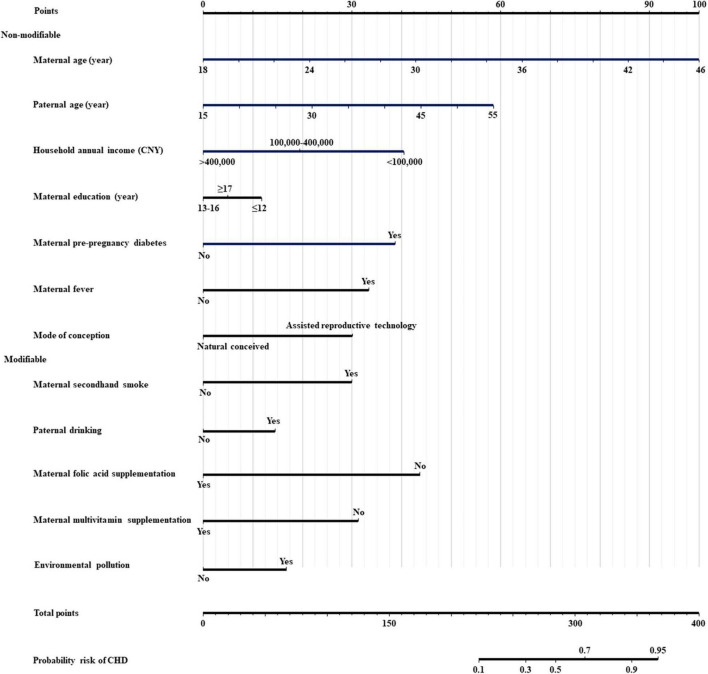
Nomogram for predicting the risk of offspring congenital heart diseases. CNY, china yuan; CHD, congenital heart disease; Modifiable, modifiable factors; Non-modifiable, non-modifiable factors.

Twelve dominant predictors, including maternal age, paternal age, household annual income, maternal education, maternal secondhand smoke exposure, paternal drinking, maternal pre-pregnancy diabetes, maternal fever, maternal folic acid supplementation, maternal multivitamin supplementation, mode of conception, and environmental pollution, were used to construct the prediction model and nomogram. There was no outlier with a model covariate of approximately ± 0.05 (Supplementary Figure 1). The variance inflation factor values of the twelve covariates ranged from 1.10 to 2.06, indicating no multicollinearity in the model (Supplementary Table 4). The predictive ability of each independent predictor of the model for CHD is reflected in the nomogram (Figure 2).

### Nomogram Validation and Application

**FIGURE 3:**
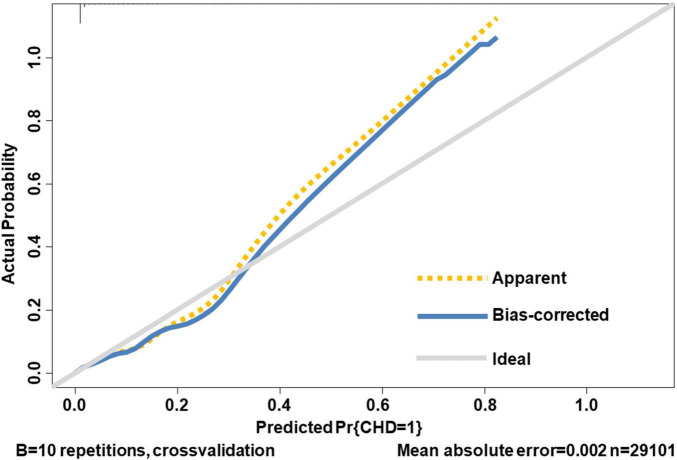
The calibration curve of internal validation. The 45-degree long gray solid line represents an ideal prediction, the brown dotted line represents the current nomogram we constructed, and the blue solid line is the bias-corrected fitted line of the nomogram using a 10-fold cross-validation method. The closer the blue solid line is to the ideal line, the better the calibration of the nomogram is.

**FIGURE 4:**
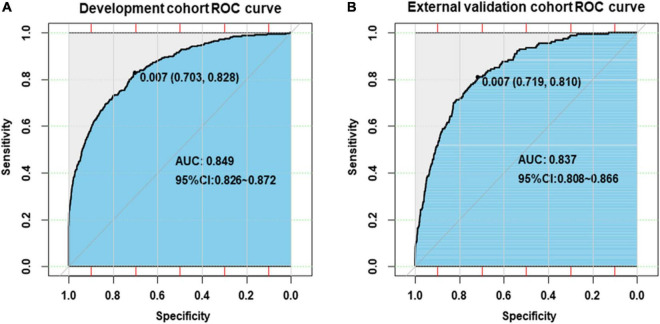
Receiver-operating-characteristic curves for the prediction of congenital heart disease by nomogram in the development cohort **(A)** and the external validation cohort **(B)**.

**FIGURE 5:**
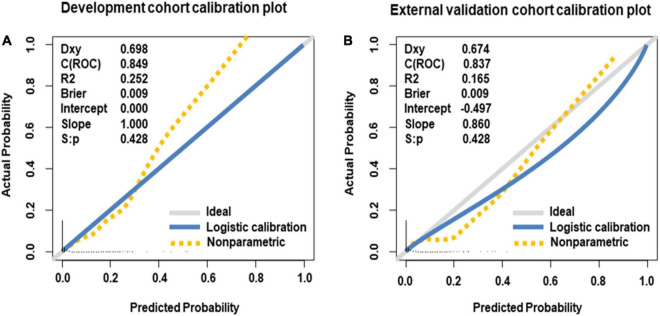
Calibration curves of nomogram for predicting offspring congenital heart disease, in the development cohort **(A)** and the external validation cohort **(B)**. The 45-degree long gray solid line represents an ideal prediction, and the blue solid line represents the predictive performance of the nomogram. The closer the blue solid line is to the ideal line, the better the predictive performance of the nomogram is.

Internal validation of the nomogram based on 10-fold cross-validation method showed the AUC of 0.843, indicating the adequate overall risk discrimination power of the nomogram. The nomogram calibration curve showed good agreement between the nomogram-predicted probability of CHD and actual presence of CHD (Figure 3). As for external validation, the nomogram showed good discriminatory power, with AUCs of 0.849 and 0.837 in the development and the external validation cohorts, respectively (Figure 4). The Delong test revealed that the difference was not statistically significant between the AUCs of the nomogram (*D* = 0.627 and *P* = 0.531), indicating the absence of potential overfitting. The nomogram also showed good calibration in both the development and the external validation cohorts (Figure 5).

The web-based nomogram provided a user-friendly access, which is available at https://postdocxiaoman.shinyapps.io/DynNomCHDapp/ or the QR code (Supplementary Figure 2), and then the users could self-evaluate the risk of offspring CHD by simply selecting or filling the values of predictors on the user interface. For example, in case of a pregnant woman, aged 36 years and her husband aged 40 years, annual household income of over 400,000 (CNY), was educated for ≥ 17 years, exposed to secondhand smoke, alcoholic husband, maternal pre-pregnancy diabetes, maternal fever, maternal folic acid supplementation, maternal multivitamin supplementation, conceived through assisted reproductive technology, and not exposed to environmental pollution, corresponding to points of 64 + 36 + 0 + 5 + 30 + 12 + 38 + 33 + 0 + 0 + 30 + 0 = 248, the risk probability for offspring CHD was 0.226. The screenshots of the web-based nomogram are presented in Supplementary Figures 3–5.

## Discussion

The results of this study further revealed the non-genetic causes of offspring CHD. The web-based nomogram could potentially be utilized as a convenient and effective tool for quantifying the individual risk of CHD in clinical practice.

We found that certain sociodemographic factors were dominant predictors for offspring CHD, of which the increased maternal and paternal age were risk factors (11), and a higher household annual income and maternal education level were protective factors. Joinau et al. (12) found that the increased paternal age had a higher risk of offspring CHD (summary OR, 1.16; 95% CI, 1.07–1.25) based on a meta-analysis of 9,917,011 participants, which is consistent with our study. The same study also explained that the increased paternal age has been associated with the decreased quality of semen, and increased percentage of short telomeres, especially among those aged over 34–35 years. The study of Peyvandi et al. (31) supported the inverse association between the higher household annual income and offspring CHD risk. Poverty and a low education level limited the access to comprehensive health care service, which may be related to higher CHD risk in offspring. Qu et al. (13) reported 48% and 17% higher risks of CHD in offspring whose mothers have been educated for less than 12 years, and household income is less than 3,000 CNY/person/month. Interestingly, mothers who have been educated for 13–16 years showed a stronger protective effect against offspring CHD compared with those who have been educated for more than 17 years in this study, which may be attributed to excessive stress such as work-related stress during pregnancy of highly educated mothers (32).

Besides, we also found that adverse lifestyle behaviors and environments including maternal secondhand smoke exposure (17), paternal drinking, and environmental pollution increased the risk of offspring CHD. The association between secondhand smoke exposure and offspring CHD may be explained by the fact that carbon monoxide and nicotine induce maternal and fetal circulatory endothelial dysfunction, reduced blood flow to the placenta, and interruption of cardiac angiogenesis, affecting the development of fetal heart and the function of cardiomyocytes and aortic muscle cells (33). Nie et al. (14) confirmed that paternal drinking was associated with an increased risk of CHD (adjusted OR, 2.87; 95% CI: 2.25–3.65). Alcohol may cause damage to cardiac neural crest cells, and increase apoptosis and/or hamper migration, which are critical stages of cardiac early development (34). Yang et al. (18) proved that environmental pollution was associated with the increased CHD risk in offspring. Pregnant women who were exposed to environmental pollution showed increased markers of oxidative stress, which have been reported to be related to outflow tract defects (35). Bove et al. (35) indicated that ambient particulates in the maternal residential area could be transported to the fetuses (36).

In addition, we revealed that maternal pre-pregnancy diseases and the current pregnancy information were also associated with offspring CHD. Specifically, maternal pre-pregnancy diabetes, maternal fever, and assisted reproductive technology served as risk factors, and maternal folic acid supplementation and maternal multivitamin supplementation were protective factors. Wu et al. (15) indicated that maternal pre-pregnancy diabetes was associated with a greater risk of cyanotic CHD (adjusted RRs, 4.61; 95% CI: 4.28–4.96), which was in agreement with our results. These findings highlight the important role of counseling and the management of pre-pregnancy diabetes in the prevention of CHD. Previous studies on the association between maternal fever and CHD were controversial. Yang et al. (16) systematically reviewed 16 studies involving 183,563 participants and reported an association between the maternal fever during pregnancy and greater risk of CHD in offspring (OR, 1.45; 95% CI: 1.21–1.73), whereas a recent cohort study of 77,344 pregnant women failed to conclude such a relationship (21). In this study, maternal fever was a risk factor for offspring CHD, the possible explanation may be that fever could activate temperature-sensitive ion channels of neural crest cells in fetuses, resulting in cardiac defects (37). The association between maternal folic acid as well as multivitamin supplementation and the risk of offspring CHD was controversial (19, 22, 23). The protective effect of maternal folic acid and multivitamin supplementation against offspring CHD may be because folic acid and multivitamin have antagonistic effects on the process of vascular disruption, and have a rescue effect on apoptotic cells with folic acid deficiency (6). Offspring of our study conceived through assisted reproductive technology was more likely to develop CHD compared with those conceived spontaneously (20). Wang et al. supported our view and explained that fetuses conceived through assisted reproductive technology carried more germline *de novo* mutations than those conceived spontaneously, which could affect protein-coding genes and further result in a higher risk of CHD (38).

Previous studies on the prediction model of CHD were mainly focused on predicting the risk of adverse maternal or neonatal outcomes in pregnant women with CHD (39, 40). There are few studies on the development of the offspring CHD prediction model based on parental non-genetic and environmental factors. Liang et al. (6) built an offspring CHD prediction model using respiratory infections, water pollution, adverse emotions, and nutrition supplementation based on data from a single center involving 654 participants and reported an AUC of 0.72 (95% CI: 0.68–0.76). Our nomogram showed better discriminatory power with AUCs of 0.849 and 0.837 in the development and the external validation cohorts, respectively. Besides, the web-based nomogram developed in this study could quantify the individual risk of CHD and is freely available to users; it could provide women with affected fetuses reproductive choices (*i.e.*, termination of pregnancy, continuation of pregnancy, or give up for adoption) at the earliest opportunity.

### Implications for Clinical Practice

In China, the current recommendation is to offer second-trimester ultrasound examination to screen for CHD and other malformations. An effective screening method for CHD in the first trimester is still lacking. Our study has demonstrated that the nomogram achieves good performance in the development and external validation cohorts. This implies that, if the nomogram is used, pregnant women with high-risk of offspring CHD could be detected in the early stage of pregnancy, and more preventive interventions could be provided to them as early as possible. Therefore, screening of pregnant women during routine first-trimester prenatal examination to identify those with high risk of offspring CHD should be encouraged. In addition, most of the predictors included in our nomogram are easily accessible and modifiable. Healthcare workers and expectant parents should be educated about the risk and protective factors of CHD, and the corresponding study should also be conducted to promote the awareness of influences from non-genetic factors in social communities.

### Strengths and Limitations

This study has several strengths. First, our study has a large sample size based on a nationwide multicenter study, which enabled us to build a nomogram with a small expected relative bias. Second, detailed information of participants was recorded, allowing us to analyze potential predictors as many as possible, including demographic characteristics, lifestyle behaviors, environmental pollution, maternal disease history, and the current pregnancy information of the study population. Third, the nomogram established shows good performance, the web-based nomogram could be conveniently and effectively applied to the prediction of CHD, especially in developing countries that lack well-developed pregnancy monitoring systems. Fourth, we conducted extensive internal and external validations using population from different centers based on TRIPOD criteria, which increases the generalizability of the findings.

However, we acknowledge that several limitations cannot be neglected. First, although we included as many predictors as possible in this study, we cannot rule out the possibility of some residual effects related to other factors such as the adverse emotion of pregnant women during pregnancy (32). Second, participants with outliers or missing data in any variable of interest were excluded, which may have contributed to bias in our findings, but we believe that our final conclusion is robust since the total rate of outliers and missing data is only 0.35% (160 of 45,504). Third, maternal/paternal data such as drinking, smoking, and environmental pollution were from self-administered questionnaire, which may decrease the reliability of our findings. However, the questions are presented in simple language and have been piloted, and the forms completed by participants of the study and medical staff have shown high concordance. Fourth, our participants were predominantly from a single ethnicity and tertiary hospitals, and the nomogram requires prospective validation in other ethnicities and general community populations. Fifth, the future use of our nomogram may be limited by the annual increase in household income like in 10 years when people have much higher income, so updating periodically is needed. The nomogram may not be applicable to individuals with factors lying outside the range of the nomogram such as younger or older pregnant women.

## Conclusion

In this study, we have revealed dominant parental predictors and presented a web-based nomogram, which could potentially be utilized as a convenient and effective tool for quantifying the individual risk of CHD and promptly identifying high-risk population. Healthcare workers and expectant parents should be educated about the risk and protective factors of CHD, and the corresponding study should also be conducted for government to improve their education and restrict the number of smelly water ditches, garbage stations, or coal-fired factories near the residential area.

## Data Availability Statement

The original contributions presented in the study are included in the article/Supplementary Material, further inquiries can be directed to the corresponding authors.

## Ethics Statement

The studies involving human participants were reviewed and approved by the Ethical Committee of Beijing Obstetrics and Gynecology Hospital, Capital Medical University (number: 2018-KY-003-01). The patients/participants provided their written informed consent to participate in this study.

## Author Contributions

WY had full access to all the data of the study and took responsibility for the integrity and accuracy of the data analysis. WY, CY, G-HD, WZ, MZ, and YS contributed to concept and design. MZ, YS, WZ, WY, CY, G-HD, B-YY, GC, XZ, and RL contributed to acquisition, analysis, or interpretation of data. MZ, YS, WY, CY, G-HD, and WZ contributed in drafting the manuscript. MZ, YS, and WZ contributed to statistical analysis. WY, CY, MZ, and YS obtained funding. WY, CY, G-HD, and WZ contributed to supervision. All authors contributed to administrative, technical, and material support and contributed to critical revisions of the manuscript for important intellectual contents.

## Conflict of Interest

The authors declare that the research was conducted in the absence of any commercial or financial relationships that could be construed as a potential conflict of interest.

## Publisher’s Note

All claims expressed in this article are solely those of the authors and do not necessarily represent those of their affiliated organizations, or those of the publisher, the editors and the reviewers. Any product that may be evaluated in this article, or claim that may be made by its manufacturer, is not guaranteed or endorsed by the publisher.

## Footnotes

1
http://www.rproject.org
