# Supplementary material for: How Parental Predictors Jointly Affect the Risk of Offspring Congenital Heart Disease: A Nationwide Multicenter Study Based on the China Birth Cohort
Source: Front Cardiovasc Med. 2022 Jun 3;9:860600. doi: 10.3389/fcvm.2022.860600 (PMC9204142; doi:10.3389/fcvm.2022.860600)
Supplement: Supplementary file 1 [file Data_Sheet_1.pdf]

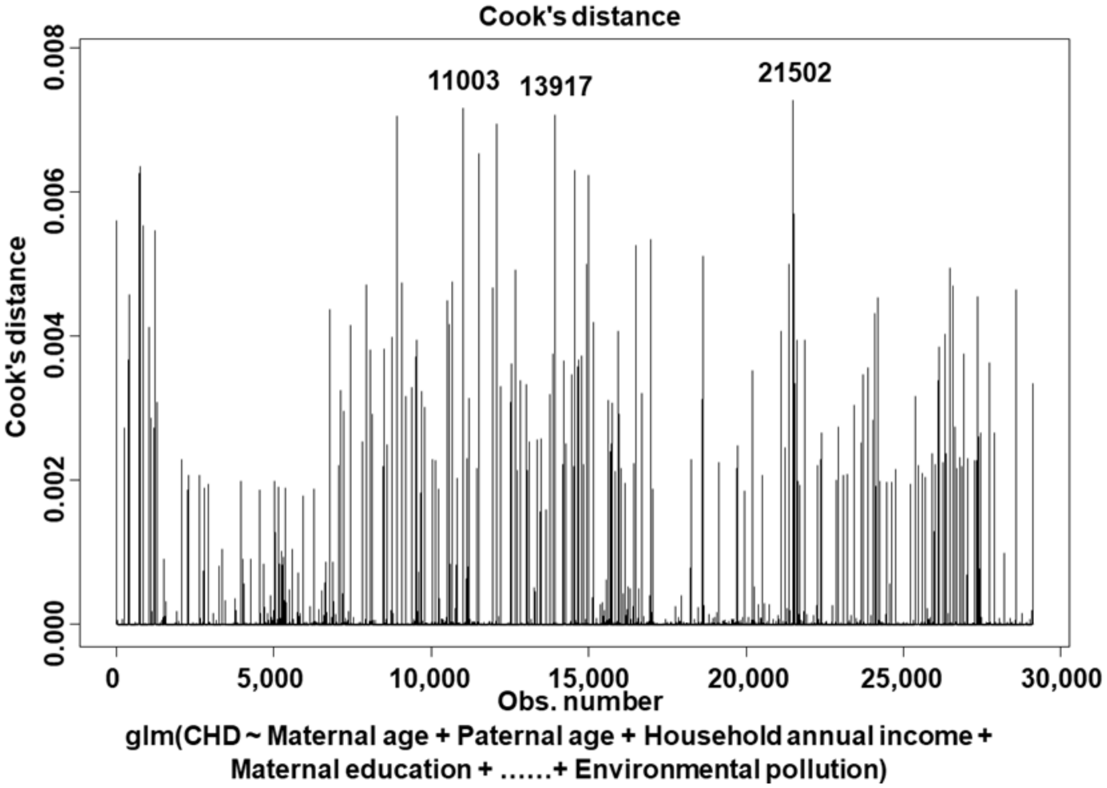

FIGURE S1 | Model outlier diagnosis. The absolute value of the cook's distance is between ± 1, thus indicating that the model does not have an outlier. CHD, congenital heart disease.

FIGURE S2 | The 'QR code' of the web-based nomogram.

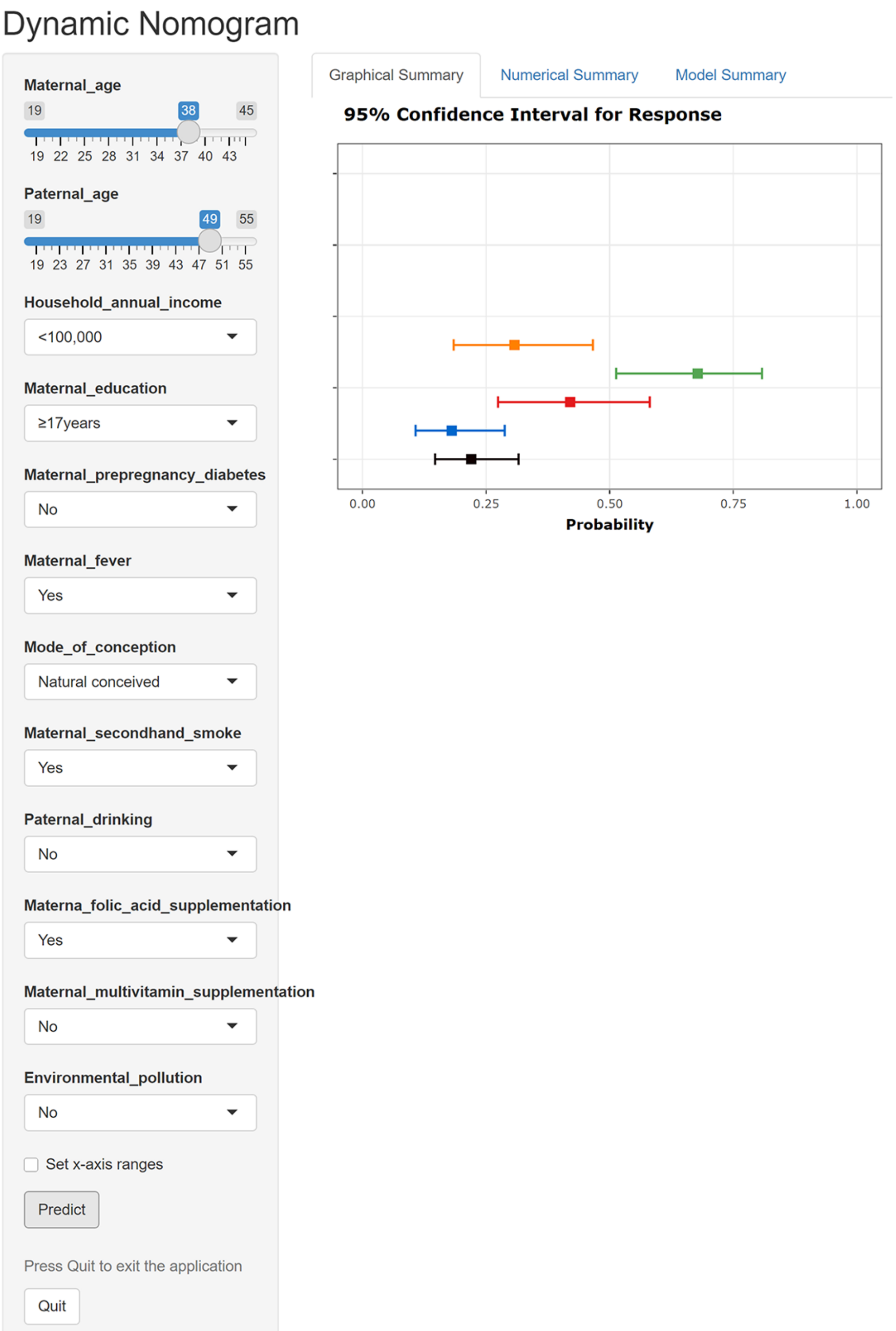

FIGURE S3 | The ‘Graphical Summary’ tab of the web-based nomogram.

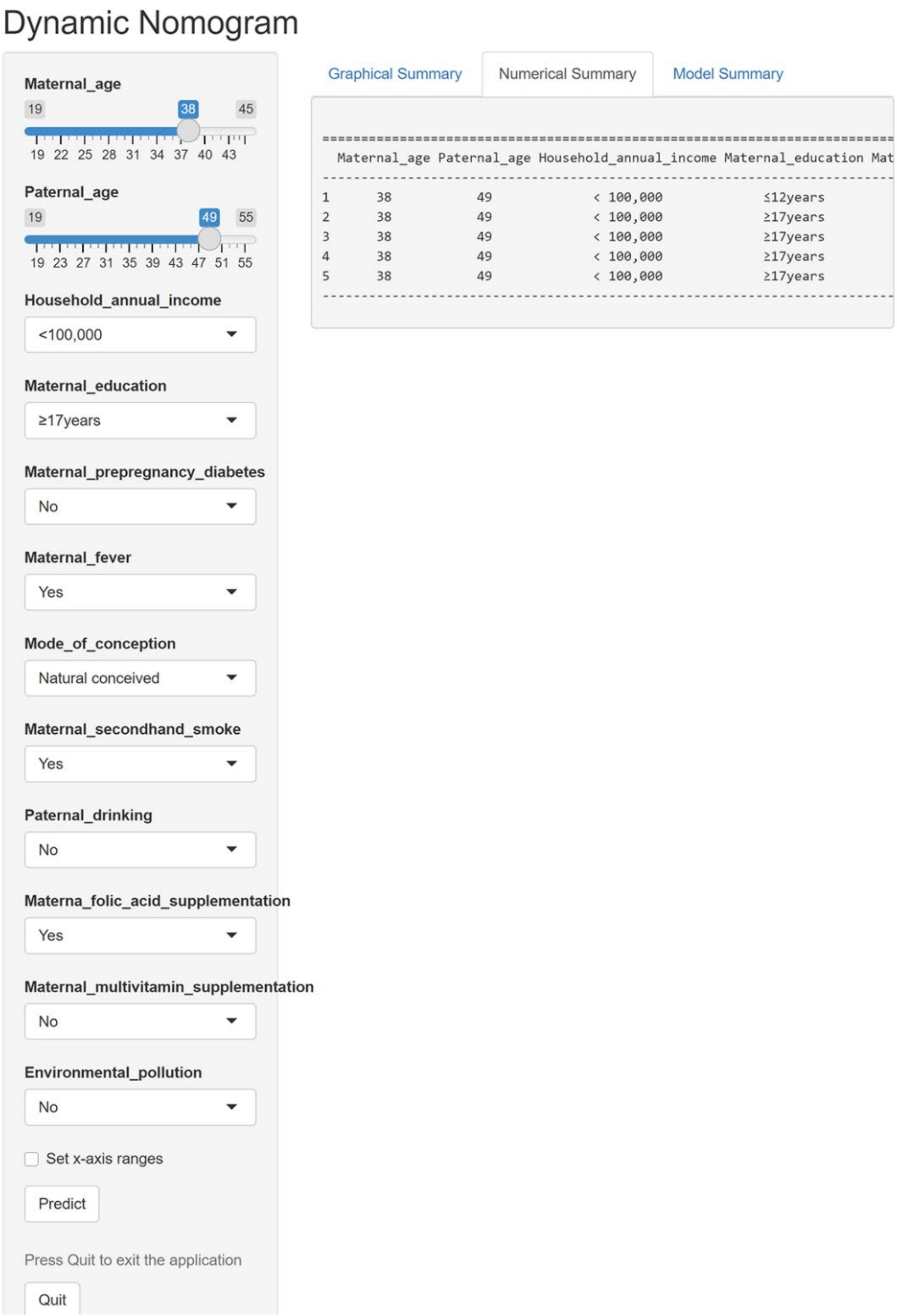

FIGURE S4 | The ‘Numerical Summary’ tab of the web-based nomogram.

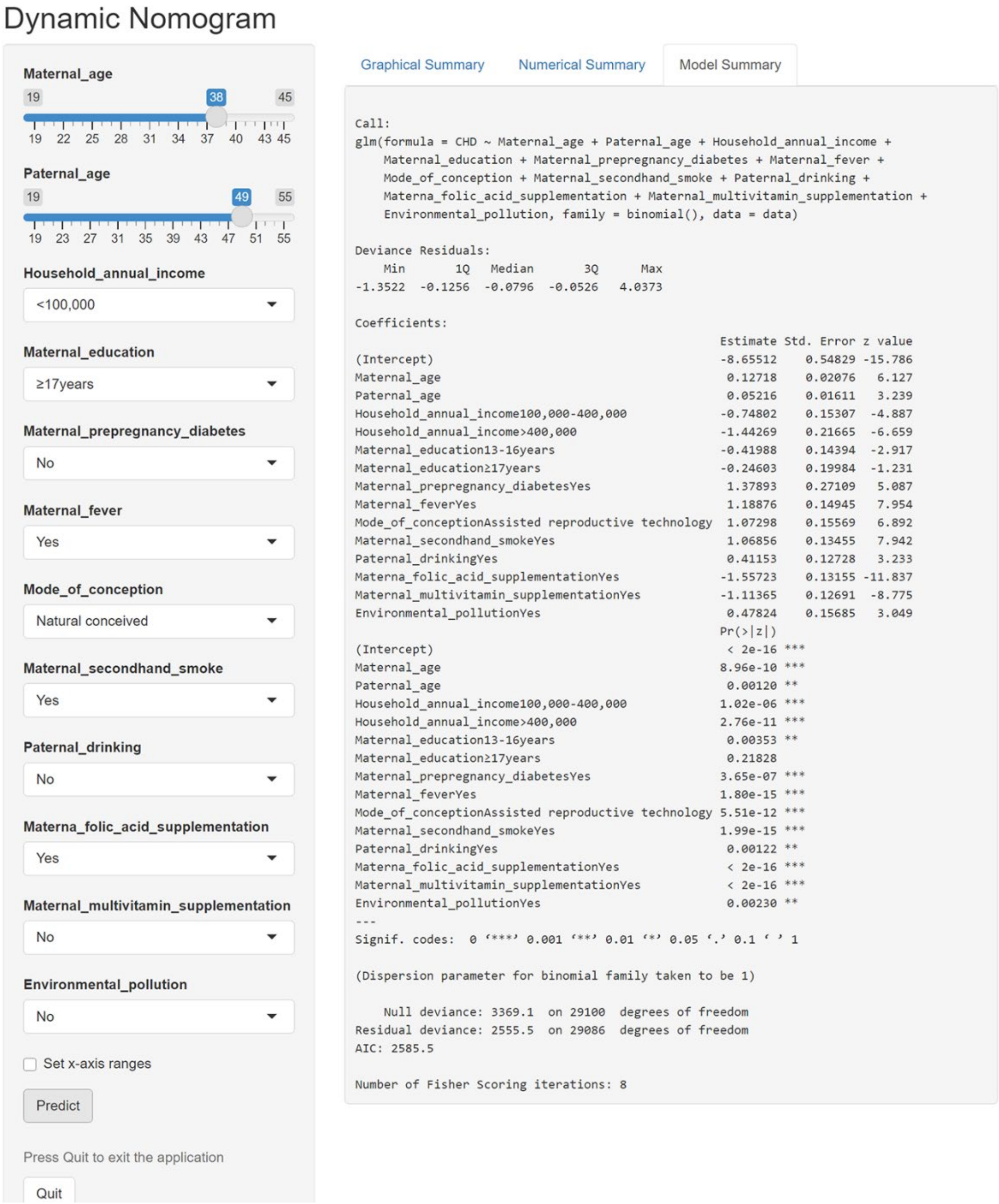

FIGURE S5 | The 'Model Summary' tab of the web-based nomogram.
