# Supplementary material for: How Parental Predictors Jointly Affect the Risk of Offspring Congenital Heart Disease: A Nationwide Multicenter Study Based on the China Birth Cohort
Source: Front Cardiovasc Med. 2022 Jun 3;9:860600. doi: 10.3389/fcvm.2022.860600 (PMC9204142; doi:10.3389/fcvm.2022.860600)
Supplement: Supplementary file 2 [file Table_1.DOCX]

**TABLE S1 |** Hospitals of the Development and the External Validation Cohorts.

| **Hospital** | **Hospital level** | **Region** |
| --- | --- | --- |
| Development cohort |  |  |
| Beijing Obstetrics and Gynecology Hospital, Capital Medical University | Grade-A Tertiary Obstetrics and Gynecology Hospital | Eastern China |
| A | Grade-A Tertiary Obstetrics and Gynecology Hospital | Eastern China |
| B | Grade-A Tertiary general Hospital | Eastern China |
| C | Grade-A Tertiary general Hospital | Eastern China |
| External validation cohort |  |  |
| D | Grade-A Tertiary Obstetrics and Gynecology Hospital | Central China |
| E | Grade-A Tertiary Obstetrics and Gynecology Hospital | Central China |
| F | Grade-A Tertiary Obstetrics and Gynecology Hospital | Western China |
| G | Grade-A Tertiary Obstetrics and Gynecology Hospital | Western China |
| H | Grade-A Tertiary Obstetrics and Gynecology Hospital | Western China |
