# Supplementary material for: How Parental Predictors Jointly Affect the Risk of Offspring Congenital Heart Disease: A Nationwide Multicenter Study Based on the China Birth Cohort
Source: Front Cardiovasc Med. 2022 Jun 3;9:860600. doi: 10.3389/fcvm.2022.860600 (PMC9204142; doi:10.3389/fcvm.2022.860600)
Supplement: Supplementary file 4 [file Table_3.docx]

**TABLE S3 |** Type of Congenital Heart Disease.

| **Congenital Heart Disease Type** | **ICD Code** | **n (%)** |
| --- | --- | --- |
| Ventricular septal defect | Q21.000 | 163 (35.75) |
| Multiple congenital heart diseases | ­­— | 119 (26.10) |
| Tetralogy of Fallot | Q21.300 | 61 (13.38) |
| Atrial septal defect | Q21.100 | 21 (4.61) |
| Left superior vena cava | Q26.100 | 16 (3.51) |
| Patent ductus arteriosus | Q25.000 | 14 (3.07) |
| Atrioventricular septal defect | Q21.200 | 13 (2.85) |
| Corrected transposition of great arteries | Q20.301 | 12 (2.63) |
| Single ventricle | Q20.401 | 9 (1.97) |
| Double outlet right ventricle | Q20.100 | 9 (1.97) |
| Double aortic arch | Q25.402 | 5 (1.10) |
| Congenital pulmonary valve stenosis | Q22.100 | 4 (0.88) |
| Pulmonary atresia | Q25.500 | 4 (0.88) |
| Ebstein anomaly | Q22.500 | 3 (0.66) |
| Complete transposition of great arteries | Q20.302 | 3 (0.66) |

*ICD, International Classification of Diseases.*
