# Supplementary material for: How Parental Predictors Jointly Affect the Risk of Offspring Congenital Heart Disease: A Nationwide Multicenter Study Based on the China Birth Cohort
Source: Front Cardiovasc Med. 2022 Jun 3;9:860600. doi: 10.3389/fcvm.2022.860600 (PMC9204142; doi:10.3389/fcvm.2022.860600)
Supplement: Supplementary file 5 [file Table_4.docx]

**TABLE S4 |** Variance Inflation Factor Values of Twelve Covariates.

| **Covariate** | **GVIF** | **Df** | **GVIF^(1/(2*Df))** |
| --- | --- | --- | --- |
| Maternal age, year | 2.06 | 1 | 1.44 |
| Paternal age, year | 2.03 | 1 | 1.43 |
| Household annual income, CNY | 1.26 | 2 | 1.06 |
| Maternal education | 1.25 | 2 | 1.06 |
| Maternal secondhand smoke exposure | 1.07 | 1 | 1.04 |
| Paternal drinking | 1.05 | 1 | 1.02 |
| Maternal pre-pregnancy diabetes | 1.09 | 1 | 1.05 |
| Maternal fever | 1.11 | 1 | 1.05 |
| Maternal folic acid supplementation | 1.04 | 1 | 1.02 |
| Maternal multivitamin supplementation | 1.06 | 1 | 1.03 |
| Mode of conception | 1.11 | 1 | 1.05 |
| Environmental pollution | 1.10 | 1 | 1.05 |

*GVIF, Generalized variance inflation factor;* *Df, degree of freedom; CNY, China Yuan.*
